# Learning from a clinical microsystems quality improvement initiative to promote integrated care across a falls care pathway

**DOI:** 10.1017/S1463423618000567

**Published:** 2018-08-23

**Authors:** Kate Gerrish, Carol Keen, Judith Palfreyman

**Affiliations:** 1 Emeritus Professor of Nursing, School of Nursing and Midwifery, University of Sheffield, Sheffield, UK; 2 Lead Therapist, Pulmonary Vascular Diseases Unit, Sheffield Teaching Hospitals NHS Foundation Trust, Sheffield, UK; 3 Trial Co-ordinator, Faculty of Nursing, University of Alberta, Canada

**Keywords:** case study, clinical microsystems, falls care pathway, integration acute and community services, quality improvement

## Abstract

**Aim:**

To identify learning from a clinical microsystems (CMS) quality improvement initiative to develop a more integrated service across a falls care pathway spanning community and hospital services.

**Background:**

Falls present a major challenge to healthcare providers internationally as populations age. A review of the falls care pathway in Sheffield, United Kingdom, identified that pathway implementation was constrained by inconsistent co-ordination and integration at the hospital–community interface.

**Approach:**

The initiative utilised the CMS quality improvement approach and comprised three phases. Phase 1 focussed on developing a climate for change through engaging stakeholders across the existing pathway and coaching frontline teams operating as microsystems in quality improvement. Phase 2 involved initiating change by working at the mesosystem level to identify priorities for improvement and undertake tests of change. Phase 3 engaged decision makers at the macrosystem level from across the wider pathway in achieving change identified in earlier phases of the initiative.

**Findings:**

The initiative was successful in delivering change in relation to key aspects of the pathway, engaging frontline staff and decision makers from different services within the pathway, and in building quality improvement capability within the workforce. Viewing the pathway as a series of interrelated CMS enabled stakeholders to understand the complex nature of the pathway and to target key areas for change. Particular challenges encountered arose from organisational reconfiguration and cross-boundary working.

**Conclusion:**

CMS quality improvement methodology may be a useful approach to promoting integration across a care pathway. Using a CMS approach contributed towards clinical and professional integration of some aspects of the service. Recognition of the pathway operating at meso- and macrosystem levels fostered wider stakeholder engagement with the potential of improving integration of care across a range of health and care providers involved in the pathway.

## Introduction

This development paper reports on the learning to arise from an innovative approach to developing an integrated falls pathway across community and acute services using the clinical microsystems (CMS) methodology developed by the Dartmouth Institute, USA. The initiative was a partnership with Sheffield Teaching Hospitals NHS Foundation Trust, which encompasses acute and community services in the city, and other service providers involved in the falls pathway. The National Institute for Health Research (NIHR) Collaboration for Leadership in Applied Health and Research and Care (CLAHRC) for South Yorkshire worked with the project team to capture the learning arising from the initiative.

### Background

Falls pose a significant challenge internationally to ageing populations with increasingly high expectations of active living. In the United Kingdom, people aged 65 and older are at most risk of falling, with a third of people older than 65 years and half of people older than 80 years falling at least once per year [National Institute for Health and Care Excellence (NICE), 2013]. Moreover, people who have fallen once are at greater risk of falling again within the following 12 months (Lord *et al*., 2001). Falls impact on patients, families, carers and communities. The financial costs from fall-related injuries are substantial: the healthcare costs associated with falls in the United Kingdom are estimated to be in excess £2.3 billion per year (NICE, 2013). The wider costs to society are likely to be far greater.

The reasons why people fall are complex and influenced by contributing factors such as physical illness, cognitive impairment, side effects of medication, problems with balance and mobility, and increasing age (NICE, 2013). National and international guidelines recommend multi-factorial assessment and integrated management targeted at effective prevention and care for this group of patients (World Health Organisation, 2007; NICE, 2013). One approach to delivering this is through care pathways.

Care pathways bring together multi-disciplinary decision-making regarding the care of a well-defined group of patients over a specific time period to enhance patient care. They should deliver, by definition, integrated care across community and hospital services. The processes, flows and outcomes of the pathway are established through multi-professional collaboration, bringing together research evidence and clinical consensus (Lodewijckx *et al*., 2012). Their key characteristics include communication and co-ordination among team members, patients and families (Schrijvers *et al.*, 2012).

Interprofessional collaboration, including overlapping of roles, shared decision-making and problem-solving, is critical in delivering care pathways (Hartgerink *et al.*, 2013). Wodchis *et al*. (2015) found that successful integration benefits from local leadership and vision, good communication and relationships, and common organisational structures. Hard work is required to overcome pre-existing boundaries. They saw benefits in bottom-up local initiatives to develop pathways, supported by higher-level stimulus and structures.

It is against this backdrop that we undertook an initiative to address quality improvement within the falls pathway across Sheffield, a UK city with a population of over 500 000 people. The Sheffield Falls Care Pathway was first developed in 2010 and comprised a three-tiered level of assessment and intervention spanning a range of acute and community services (see Table 1).

**Table 1:** Sheffield falls care pathway 2010

Level	Activity	Staff involved
Level 1	Initial screening assessment	Undertaken opportunistically by community-based staff in health and social care
Level 2	Completion of multi-factorial falls risk assessment, and appropriate intervention	Therapy and nursing staff across a number of community and acute services, some dedicated to falls prevention and management, others targeted at a wider population groups
Level 3	Hospital-based multi-disciplinary model for diagnosis of unexplained falls	Referral initiated medical diagnostic assessment

In April 2011, Sheffield’s adult community healthcare services, including domiciliary nursing and community therapy services combined with Sheffield Teaching Hospitals NHS Foundation Trust (STH), form a single healthcare provider. This integration of adult acute and community services provided an opportunity to review and enhance the existing falls care pathway.

In June 2012, STH and local healthcare partners began an initiative to train staff to become coaches in the CMS approach to quality improvement (Dartmouth Institute, 2011). This is a structured approach to quality improvement that embodies a bottom-up approach to delivering change. A CMS is a small, functional, frontline unit that delivers healthcare, for example an outpatient falls clinic, an inpatient orthopaedic ward or a community healthcare practice. The CMS approach is based on the assumption that the quality of care provided by a health system can be no better than that delivered by the functional units of which the system is composed. The CMS methodology is designed to enable transformation of care through engaging frontline multi-professional healthcare staff in the microsystem, along with patients and carers (Nelson and Batalden, 2003).

The CMS approach is based on a framework of ‘5P’s’ – purpose, patients, professionals, processes and patterns. Analysis of these components of the microsystem effects change by progressing through steps that include process mapping, measurement, tests of change and establishing sustained improvement. Although the CMS is an important unit through which quality improvement can be addressed, it does not operate in isolation. It sits within a complex multi-level context, comprising the broader macrosystem (health and social care system) and the mesosystem (organisation) that impact on the work undertaken at the microsystem. Addressing meso- and macrosystem priorities, systems and processes can support microsystem quality improvement (Godfrey *et al*., 2008).

### Problem statement

A review of falls in Sheffield undertaken in December 2011 identified that the hip fracture rate resulting from falls was 17% higher than the national average (Sheffield City Council, 2011). More recently, Age UK Sheffield identified that 39 651 people aged over 50 attended Sheffield hospitals after suffering a fall during 2013. (http://www.sth.nhs.uk/news/news?action=view&newsID=499). Preventing older people from falling, as well as ensuring integrated services posed challenges for healthcare providers and local authorities in Sheffield. Whereas hospital, community and the local authority provided falls services, the number of people accessing these services was low in comparison to those who had the capacity to benefit from them. Additionally, successful implementation of the pathway was constrained by inconsistent co-ordination and integration at the community–hospital interface. Access to the pathway was unclear with a referral overlap by different healthcare professionals.

The Sheffield Falls Care Pathway could be viewed as a series of interconnected microsystems located within different areas (community and hospital) of the large organisation (mesosystem) and spans different organisations (macrosystem). This understanding of the pathway combined with Sheffield’s investment in training microsystem coaches suggested that the CMS approach had the potential to deliver care pathway quality improvement.

## Overview of the falls quality improvement initiative

The quality improvement initiative ran from September 2012 to March 2015.

The aims of the initiative were to:

1. Develop an integrated community and hospital falls service designed by staff to optimise patient-centred evidence-based care in falls prevention and management.
2. Build capacity among healthcare professionals to use the CMS quality improvement methodology to support continuous quality improvement.

The project team comprised a project lead (a nurse with extensive experience of older people’s services) and three facilitators (a community matron and two physiotherapists with experience of falls services). The initiative was overseen by a consultant geriatrician with responsibility for falls services and service improvement, and by a senior member of the service improvement team.

An independent evaluation team comprising two academic/healthcare staff worked with the project team to capture learning. This involved undertaking focus group discussions and individual interviews with members of the team and observation of a range of meetings in each phase of the initiative. Interview/focus group transcripts and field notes of meetings were analysed by the evaluation team and the findings discussed with the project team in order to draw out the learning. The aim of this paper is to report on the learning to arise from using the CMS approach to promote integrated care across the falls care pathway.

The initiative comprised three phases that are described below.

### Phase 1: Developing a climate for change

The first phase focussed on training, information gathering and networking. The project team sought to develop a climate of change as a platform for work in later phases of the initiative.

#### Development of CMS coaching skills in the project team

At the outset of the initiative, the four team members attended a two-day course in CMS methodology and improvement science at the Dartmouth Institute for Health Policy and Practice in Vermont, USA. This initial training was followed by monthly remote training sessions continuing until January 2013. The course provided the team with the knowledge and skills required to begin coaching microsystem teams, which they developed further as they gained coaching experience.

#### Identifying the existing pathway and engaging key stakeholders

The team met with individual stakeholders to gain an in-depth understanding of falls services across the city, including primary and secondary healthcare providers, care agencies and ambulance services. They also liaised with agencies across the wider pathway to increase understanding of falls services, including general practitioners and care home managers.

A process mapping exercise carried out in December 2012 involved representatives from services in all three levels across the pathway. This identified several challenges in developing an integrated pathway, including:

- unclear and overlapping referral processes;
- stakeholders lacked understanding of services outside of their own, and referral processes, resulting in inappropriate referrals to some parts of the service;
- services appeared fragmented and uncoordinated;
- poor communication between different services.

##### Coaching CMS across the falls care pathway

The team worked with stakeholders to identify four areas in Level 2 services in which they initiated CMS quality improvement activities. Three discrete community-based services that covered most Level 2 services in the pathway were identified: Community Intermediate Care Services (CICS), the community-based Falls Prevention Team and the Front Door Response Team. Two CMS were initiated within CICS and one in each of the other two services. Beginning in March 2013, each area established a CMS group comprised of between four and ten frontline staff, including nurses, therapists and administrative staff. These groups met regularly (weekly or fortnightly) for 1 h with a project team member acting as a coach to each CMS.

Under the guidance and facilitation of their coach, the groups began the CMS process. This provided a systematic approach that allowed teams to develop an understanding of their work and its processes, specify aims, prioritise change ideas and run rigorous tests of change within the system. Each CMS identified and developed areas for change specific to their work, including team working and communication, use of technology to improve patient handovers and standardisation of processes for booking patient transport.

### Phase 2: Initiating change

Phase 2 began in April 2013 during a reconfiguration of community services, which included the Level 2 falls services and extended until March 2015. It built upon the initial focus on individual microsystems in Phase 1 to focus on the mesosystem comprising the STH components of the falls pathway in community and acute settings.

#### Establishing a functional mesosystem group

The Falls Project Group (FPG) was established as a functional group operating at mesosystem level to achieve change across the falls pathway. This multi-professional group included representatives from the four CMS established in Phase 1, as well as clinical managers and senior clinical staff from across the falls services.

#### Identifying priorities and undertaking tests of change

The FPG was not a microsystem as it was not based on a single frontline clinical unit. Rather it was a mesosystem group, which drew on the CMS methodology for its approach to undertaking change. The team facilitated the fortnightly meeting of this group. The FPG reviewed different components of the pathway as a whole and identified key priorities for improvement. They initiated small tests of change and used the plan–do–study–act cycle to pilot, refine and embed change within the service. These changes included:

- redesign of patient information;
- enhanced activity data collection;
- improved engagement with staff in the emergency department;
- redesigning the referral process from Level 2 to Level 3 services to avoid referral back to the general practitioner.

### Phase 3: Achieving change

Phase 3 commenced in June 2014 and engaged key decision makers in taking forward and achieving change initiated in earlier phases of the initiative.

#### Macrosystem engagement

It became apparent in Phase 2 that while the work conducted to-date had delivered valuable change and considerable momentum, several of the priorities for change identified required the engagement of more senior decision makers from the different organisations in the pathway. This need, and a concurrent organisational decision to review key pathways in newly merged services, resulted in the creation of the Falls Pathway Workstream Group (FPWG) that operated at macrosystem level. The group involved senior clinical and service managers, senior clinicians and frontline staff from across the falls pathway, as well as project team members. It built upon the work undertaken in Phases 1 and 2 and implemented a programme of transformation to enable the delivery of an integrated falls pathway across different service providers.

The FPWG undertook:

- streamlining of the referral processes;
- development of the role of a specialist community pharmacist;
- revision of falls assessment tools.

Building on foundations laid in earlier phases, and capturing the decision-making capabilities of group members, the FPWG was able to make rapid progress in implementing change. Its work continued beyond the timescale of the current initiative.

#### On-going microsystem and mesosystem working

During this phase work continued in the CMS established in Phase 1 and the FPG from Phase 2 to bring about local changes in falls services, develop networks across the pathway and provide links between the FPWG and frontline services.

## Learning from the initiative

In the following section of this paper, we reflect on progress made during the initiative and factors that influenced progress, including the CMS approach taken.

### Progress with change

The overall approach taken enabled notable progress to be made towards achieving change in relation to key aspects of the falls care pathway. Streamlining the referral process to allow direct referral from Level 2 (community) to Level 3 (hospital) services, and improving the referral process in the emergency department to the falls service led to improvements in the number of patients referred appropriately and in the timeliness of referral. Patients assessed by the community-based active recovery service are now seen within 2 h when urgent and within 48 h if non-urgent.

The waiting times for the Level 3 service were reduced and standardised goals for times from referral to assessment were agreed for urgent and non-urgent community patients. Patients referred to the service now receive home review on the week of referral and clinic assessment within two weeks. This compares favourably with average waits of six weeks, two years previously.

Streamlining the referral process from Level 2 to Level 3 services, and from the emergency department into the falls service increased the numbers of patients able to access appropriate care. Waiting times to Level 3 services were reduced. A business case was developed to develop a community pharmacist role within the pathway to review at-risk patients receiving polypharmacy.

Although the organisational changes taking place were unsettling, some aspects provided eventual opportunity for achieving the aims of the initiative. Level 3 falls services were relocated from hospital to a community base, where they sat alongside Level 2 falls services: their integration was a focus of the FPG in Phase 2. The FPWG established in Phase 3 arose in part from a realignment of services within the organisation to better reflect patient pathways.

In addition, the groups and networks established provided a platform for on-going improvement of services across the pathway. The initiative subsequently influenced the redesign of a new discharge pathway ensuring equality of access to specialist falls care by using the ‘Discharge to Assess’ approach (NHS England, 2016). Sheffield’s health and social care services applied CMS methodology to redesign the standard approach of assessing patients’ on-going care needs on completion of acute hospital care. Instead of completing this assessment in hospital, patients are transferred home for immediate assessment and provision of care. This resulted in a reduction in length of stay, more timely falls risk assessment at home and reduced inpatient falls (Offord *et al*., 2017).

### The CMS approach

CMS was a new approach to quality improvement for most staff. It required a ‘bottom-up’ approach to change in which frontline staff within the microsystem identified their own priorities for improvement (Burnes, 2009). During Phase 1, concerns arose where priorities identified in individual microsystems appeared to be at odds with organisational priorities or of the aims of the initiative itself. Significant time and effort were required from the project team to resolve these tensions.

The CMS methodology involves a long lead-time in collecting baseline data and analysing current practice before identifying priorities for change. Progress was slow. For example, one CMS took several weeks to identify the specific focus of their improvement work. This was frustrating for group members who struggled to remain engaged, and their managers who sought quicker results.

The progress made with introducing change through CMS was adversely affected by on-going reconfiguration of services in teams delivering falls prevention and by wider organisational change. The CMS from Phase 1 were affected by service reconfiguration, resulting in premature closure of three microsystems because the teams involved did not exist as they once had, or managers withdrew support due to changes taking place.

Engagement with CMS activity was adversely affected by anxiety and uncertainty amongst frontline staff as well as other stakeholders in the pathway, resulting from service review and anticipated change. Senior managers prioritised supporting and embedding service reconfiguration over the initiative. Pathway maps that were developed in Phase 1 required revision due to service change.

### Cross-boundary working at mesosystem and macrosystem levels

Whereas CMS methodology underpinned the quality improvement taken forward in Phase 1, a broader approach engaging the meso- and macrosystem levels was needed to achieve change across the pathway in Phases 2 and 3. The falls pathway operated at mesosystem level and comprised multiple microsystems. The project team needed to engage with different microsystems across the wider pathway. Frontline staff within a particular microsystem needed to learn about the work of other microsystems in the pathway and become more open to collaborative working and ideas for change. This was achieved by mapping the falls pathway, promoting collaboration and information sharing across microsystems and through the establishment and operation of the FPG.

In order to bring about meaningful change across the pathway, some activities required access to key decision makers at the macrosystem level. Occasion arose where staff working at microsystem or mesosystem level identified enhancements to the pathway, for example the development of new roles, which could not be enacted without senior support. As the initiative progressed, organisational alignment along the pathway and the setup of the FPWG facilitated improved access to key personnel.

### Enabling stakeholder engagement

The initiative successfully engaged stakeholders from across the falls pathway and wider health and care agencies associated with patients at risk of falling. The CMS approach was instrumental in facilitating engagement. CMS established in Phase 1 engaged frontline clinicians and their managers in discussions about improvements in the pathway.

Applying the principles of the CMS approach with the mesosystem FPG group resulted in an inclusive, non-threatening forum that involved senior clinicians from different professional groups and their managers as representatives from relevant areas.

The FPWG, which operated at macrosystem level, included key decision makers from a wide range of services. In addition to project team members liaising with key stakeholders, as the initiative progressed, those stakeholders engaged each other in improving services and also extended collaboration to include senior executives and health service commissioners. Groups from acute, community, health and social care were thereby represented at different stages of the initiative. In addition, the initiative reached out to services not directly involved in falls services, for example care homes and pharmacy.

Stakeholder engagement and pathway mapping led to a shared understanding of the complexity of the existing falls pathway, the lack of co-ordination of different services and problems with the referral process. An understanding of the organisation as a whole and the interface between community and hospital services led to an appreciation of the complexity of introducing change.

### Building quality improvement capability and capacity

The CMS approach enabled capability and capacity building in quality improvement across the organisation. Project team members developed expertise in coaching microsystems quality improvement and used the techniques to further develop their expertise in leading and influencing, and in negotiating between groups at micro and mesosystem levels. Frontline staff engaged in CMS teams developed a greater understanding of how they could initiate change, and a greater awareness of quality improvement and the steps involved. Moreover, they became empowered to take on improvement work themselves. They grew noticeably in confidence as they felt that more senior colleagues respected their opinions.

## Discussion

Although CMS is an established quality improvement methodology there are relatively few publications that describe how it has been used in different healthcare settings. This paper has described how the approach was applied in one setting and the learning to arise. The initiative sought to achieve greater integration of different professional groups and services across the falls care pathway. The pathway comprised a series of interconnecting microsystems spanning the organisational mesosystem and the wider macrosystem comprising health and care agencies, and the voluntary sector across Sheffield.

Fulop *et al*.’s (2005) typology of integrated care provides a useful way of examining progress made towards an integrated falls care pathway. The initiative took place at a time of vertical organisational integration at the meso-level involving community and hospital healthcare services merging to form a single organisation. It is widely recognised that organisational integration takes considerable time and energy to achieve and during the process changes to services can be slow to develop (Wodchis *et al*., 2015). Whereas the merger of the two organisations helped provide the impetus for working towards a more integrated falls care pathway, the impact of the organisational turbulence on frontline staff and managers delayed progress. Nevertheless, some progress was made towards achieving service integration, most notably in relation to streamlining of the referral process from Level 2 to Level 3 services. Clinical integration was also achieved in relation to a review of shared referral guidelines and assessment tools used across different professional groups in community and hospital settings. There was also some evidence of normative integration emerging whereby an ethos of shared commitment to co-ordinating work enabled greater collaboration between the emergency department and the community and hospital falls service.

Organisational integration at the meso-level in itself may be insufficient to overcome fragmentation of care (Curry and Ham, 2010). Arguably achieving an integrated care pathway depends less on organisational integration than on service and clinical integration. As Curry and Ham (2010) point out, the nature of multi-professional team working and the adoption of shared guidelines and policies are more likely to promote integration than the nature of organisational arrangements. Professional integration can also occur at the meso-level through partnerships within (intra) and between (inter) organisations (Valentijn *et al.*, 2013). This was evident in the current initiative whereby widespread stakeholder engagement was achieved across the pathway. This initially involved stakeholder discussions channelled through the project team, which subsequently led to stakeholder–stakeholder interaction across the pathway. This enhanced interaction became evident through the mesosystem FPG and macrosystem FPWG, where stakeholders came together to address different aspects of improving integration of care across the pathway.

The processes and interconnections of care pathways represent a highly complex system. Staff may appreciate the components local to them, but lack clarity on the wider pathway and its interactions. The project team initially used the view of the falls pathway as a series of interconnecting microsystems and as a tool to simplify and map its complexity. This mapping and visualisation of the pathway helped to develop a shared understanding of the pathway across a range of health and care stakeholders.

Simultaneously viewing the falls pathway as a mesosystem operating at an organisational level and as a macrosystem spanning different health and care agencies allowed the project team to begin to remove barriers, identify constraints and bottlenecks and improve communication and integration between the components of the pathway, working together towards a common goal. The role of the project team as CMS improvement coaches was key in delivering change across different components of the pathway and working across the boundaries of the individual elements (McKinley *et al*., 2008).

Whereas the initiative reported has focussed on using the CMS approach to promote a more integrated falls care pathway in one location, it is suggested that it may be a useful approach to use in other settings, and/or when developing or reviewing other care pathways that span community and hospital services. However, as this initiative has demonstrated, the CMS methodology is not directly applicable to quality improvement at meso- and macrosystem levels. Further thought needs to be given to how the *principles* of the CMS approach can be applied at the meso- and macrosystem levels.

## Conclusion

This initiative is set out to achieve a more integrated falls service across community and hospital services. Using a CMS quality improvement approach enabled the engagement of frontline staff from different components of the pathways and contributed towards clinical and professional integration of some aspects of the service. Viewing the pathway as a series of interrelated microsystems enabled stakeholders to understand the complex nature of the pathway and to target key areas for change. Recognition of the pathway operating at meso- and macrosystem levels fostered wider stakeholder engagement with the potential of improving integration of care across a range of health and care providers involved in the pathway. The account of the initiative provided in this paper suggests that CMS quality improvement methodology can be a useful approach to promoting integration across a care pathway.
